# Sphingolipids in Atherosclerosis: Chimeras in Structure and Function

**DOI:** 10.3390/ijms231911948

**Published:** 2022-10-08

**Authors:** Lisa Peters, Wolfgang M. Kuebler, Szandor Simmons

**Affiliations:** 1 Institute of Physiology, Charité-Universitätsmedizin Berlin, Charitéplatz 1, 10117 Berlin, Germany; 2 DZHK (German Centre for Cardiovascular Research), Partner Site Berlin, 10117 Berlin, Germany; 3 Department of Biology, Chemistry and Pharmacy, Institute of Biology, Freie Universität Berlin, Königin-Luise-Straße 1-3, 14195 Berlin, Germany; 4 The Keenan Research Centre for Biomedical Science at St. Michael’s, Toronto, ON M5B 1W8, Canada; 5 Departments of Surgery and Physiology, University of Toronto, Toronto, ON M5S 1A8, Canada

**Keywords:** cardiovascular disease, atherosclerosis, sphingolipids, ceramide, sphingosine-1-phosphate, dihydrocerammide, lactosylceramide, glucosylceramide, sphingomyelin

## Abstract

Atherosclerosis—a systemic inflammatory disease—is the number one cause of mortality and morbidity worldwide. As such, the prevention of disease progression is of global interest in order to reduce annual deaths at a significant scale. Atherosclerosis is characterized by plaque formation in the arteries, resulting in vascular events such as ischemic stroke or myocardial infarction. A better understanding of the underlying pathophysiological processes at the cellular and molecular level is indispensable to identify novel therapeutic targets that may alleviate disease initiation or progression. Sphingolipids—a lipid class named after the chimeric creature sphinx—are considered to play a critical and, metaphorically, equally chimeric regulatory role in atherogenesis. Previous studies identified six common sphingolipids, namely dihydroceramide (DhCer), ceramide (Cer), sphingosine-1-phosphate (S1P), sphingomyelin (SM), lactosylceramide (LacCer), and glucosylceramide (GluCer) in carotid plaques, and demonstrated their potential as inducers of plaque inflammation. In this review, we point out their specific roles in atherosclerosis by focusing on different cell types, carrier molecules, enzymes, and receptors involved in atherogenesis. Whereas we assume mainly atheroprotective effects for GluCer and LacCer, the sphingolipids DhCer, Cer, SM and S1P mediate chimeric functions. Initial studies demonstrate the successful use of interventions in the sphingolipid pathway to prevent atherosclerosis. However, as atherosclerosis is a multifactorial disease with a variety of underlying cellular processes, it is imperative for future research to emphasize the circumstances in which sphingolipids exert protective or progressive functions and to evaluate their therapeutic benefits in a spatiotemporal manner.

## 1. Introduction

The enigmatic character of sphingolipids has been first highlighted by assigning their name to a new class of lipids first described in 1884 by the German physician and biochemist J. L. W. Thudichum [1]. In the 1880s, he found “sphingosine” with unique chemical characteristics, which directed him to name this brain-derived lipid after the Sphinx, a mythical creature with a human head and a lion’s body. This iconic name became formative for the substance class of sphingolipids, but also adequately reflects the chimeric role of sphingolipids in the etiology of atherosclerosis. 

Cardiovascular diseases (CVDs) are the leading cause of mortality, accounting for 17.9 million deaths per year worldwide [2,3]. Atherosclerotic cardiovascular disease is a progressive and lifestyle-dependent condition characterized by arterial lesions characterized by local oxidative stress and inflammation that initiate vasoconstriction, reduced and/or turbulent flow, plaque formation, and/or hemostasis. These responses in combination with excessive plasma low-density lipoprotein (LDL) cholesterol levels, e.g., caused by poor dietary quality or sedentarism, lead to lipid deposition and atheromatous plaque formation resulting in functional and, ultimately, structural disintegrity of the arterial vessel wall [4,5,6]. This in turn triggers primary and secondary hemostasis that in combination with locally altered fluid mechanics is causally responsible for embolic complications, resulting in, e.g., myocardial infarction or ischemic strokes [7,8,9]. Therefore, a comprehensive understanding of the molecular mechanisms of disease initiation and progression is indispensable for the identification of possible therapeutic targets providing the spotlight for current atherosclerosis research.

Atherosclerosis represents a subtype of arteriosclerosis. Arteriosclerosis is the most common form of adverse vascular remodeling and is usually limited to small arteries and arterioles. This vascular remodeling comprises thickening and hardening of the arterial vessel wall, resulting in an increase in vascular stiffness and a reduction of blood flow to the tissues. All subtypes of arteriosclerosis have these processes in common, and sub-classification is solely based on the cause and localization of the vascular remodeling. Atherosclerosis—the focus of this review—describes adverse vascular remodeling stemming specifically from the formation of atherosclerotic plaques in the arteries. This plaque formation leads to thickening of the vessel wall, subsequent calcification further reduces wall compliance, and both processes together increase arterial stiffness [10,11].

**Figure 1:**
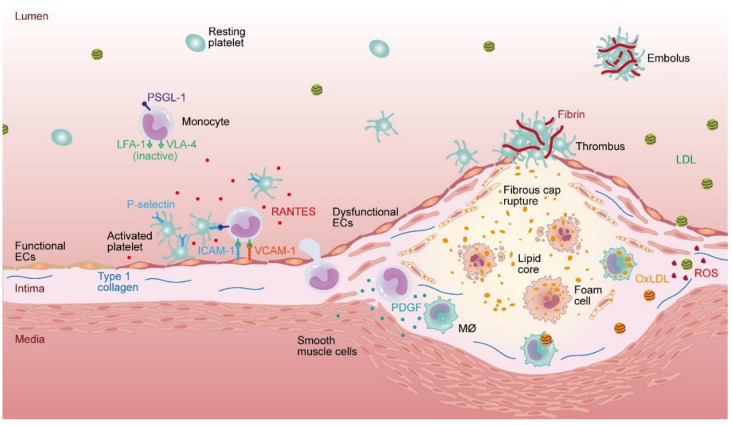
Cellular pathomechanisms of atherogenesis and progression. Environmental risk factors such as cigarette smoking and hypercaloric diet or preexisting conditions such as hypercholesterolemia, hyperglycemia or hypertension promote endothelial dysfunction and increase vascular permeability and retention of LDL in the vascular intima. Endothelial dysfunction further promotes platelet adhesion through the release of von Willebrand factor (vWF) and platelet activation by mediators such as adenosine diphosphate (ADP) and thromboxane (TxA2). Activated platelets secrete the chemokine RANTES (CCL5), which enables monocytes to adhere under flow conditions. The adhesion is further promoted by cellular adhesion molecules (CAM) expressed by activated endothelial cells. The lymphocyte function-associated antigen 1 (LFA-1) on the surface of monocytes enables their binding to intercellular adhesion molecule 1 (ICAM-1) expressed by endothelial cells. This cellular interaction is strengthened by monocytic integrin α4β1 (VLA-4) binding to vascular cell adhesion molecule 1 (VCAM-1), further mediating lateral migration and transendothelial diapedesis of monocytes into the intima. Intimal LDL is oxidized by ROS to oxidized LDL (oxLDL), which aids in the recruitment of monocytes and initiates differentiation into macrophages by scavenger receptor mediated uptake of oxLDL. Activated macrophages secrete platelet-derived growth factor (PDGF), which stimulates smooth muscle cells (SMCs) to migrate into the intima where they proliferate and produce extracellular matrix and again incorporate oxLDL. Uptake of oxLDL by SMC and macrophages leads to their differentiation into foam cells, which degrade and, in turn, release oxLDL. This self-amplifying process further attracts macrophages and SMCs that accumulate oxLDL and dying cells—the necrotic core of the atheromatous plaque. This process is accompanied by thickening of the intima limiting blood flow through the lumen and results in weakening of the fibrous cap of the vulnerable plaque. As the disease progresses, the vascular lumen becomes gradually occluded, leading to turbulent blood flow, which supports endothelial dysfunction, the expression of CAMs, and the formation of vascular lesions. Increasing instability culminates in plaque rupture and subsequent thrombus formation.

Mechanistically, the pathogenesis of atherosclerosis comprises a diversity of cell types and molecules (Figure 1). Atherogenesis is exacerbated by various environmental risk factors such as cigarette smoking and hypercaloric diet or by preexisting conditions such as hypercholesterolemia, hyperglycemia, or hypertension [12,13,14]. Underlying these risk factors is the presence of oxidative stress and, consequently, endothelial dysfunction. NO is continuously produced and released by endothelial NO synthase (eNOS) in endothelial cells at baseline. NO primarily exhibits anti-inflammatory and antithrombotic functions such as attenuation of platelet adhesion, aggregation and leukocyte adhesion [15,16]. In a stable endothelium, protective NO and harmful ROS are in balance since ROS and NO react to peroxynitrate [17]. In the progression of atherosclerosis or diabetes, eNOS produces ROS, e.g., superoxide anion instead of NO; a process also known as “eNOS uncoupling” [18]. The eNOS uncoupling further enhances superoxide anion production [19] and activation of NAD(P)H oxidase [20], which, in turn, represents a major source of the superoxide anion [20,21,22]. The subsequent imbalance toward ROS results in endothelial dysfunction, which culminates in increased vascular permeability [23,24,25] and extravasation of LDL into the intima [26,27,28]. Furthermore, endothelial dysfunction fosters platelet adhesion to von Willebrand factor (vWF) and consequential platelet activation by the release of paracrine mediators, such as adenosine diphosphate (ADP) and thromboxane (TxA2) [29]. Activated platelets also secrete the chemokine RANTES (CCL5) that immobilizes on the surface of inflamed microvascular or aortic endothelium and allows for shear-resistant monocyte arrest under flow conditions [30].

Parallel endothelial dysfunction is associated with the expression of cell adhesion molecules (CAMs), i.e., ICAM-1 and VCAM-1, on vascular endothelial cells, and subsequent tethering, rolling and adhesion of monocytes on the endothelium—a hallmark of atherogenesis [31,32,33]. The accompanying morphological change allows monocytes to transmigrate across the endothelium into the intima in a process called diapedesis. Upon activation, monocytes become synthesizers of ROS, i.e., superoxide, hydroxyl radicals, and peroxyl radicals that support protein degradation and DNA oxidation, but, most importantly, lipid peroxidation, which is a hallmark of chronic inflammatory diseases including atherosclerosis [34,35,36,37]. In this milieu, ROS can oxidize native non-atherogenic LDL to oxidized low-density lipoprotein (oxLDL). These aid in the activation of monocytes through scavenger receptor pathways, which in turn maturate to macrophages and, subsequently, cholesterol rich foam cells. The differentiation of monocytes into macrophages is a multistep process initiated by the recruitment of monocytes to the lesion site accompanied by the secretion of granulocyte–macrophage colony-stimulating factor (GM-CSF) and macrophage colony-stimulating factor (M-CSF). These factors, in turn, drive the proliferation of intimal cells in the early phase of atherosclerosis [38] and promote advanced plaque progression by increasing macrophage apoptosis susceptibility [39]. The altered transcriptional program in the activated monocytes promotes macrophage maturation [40,41]. Specifically, by expression of atherogenic scavenger receptors including CD36, macrophages become enabled to internalize oxidatively modified proteins such as oxLDL. This oxLDL uptake by CD36 promotes macrophage differentiation and foam cell formation as illustrated by the fact that apolipoprotein E (ApoE)-deficient animals that lack expression of CD36 show a marked reduction in atherosclerotic lesions as compared to ApoE-deficient mice expressing CD36 [42,43]. Due to the prevailing oxidative stress by oxLDL, smooth muscle cells (SMC) express scavenger receptors and take up oxLDL, resulting in the formation of foam cells [44,45,46,47,48]. Concomitantly, macrophages proliferate in the intima and amplify the maladaptive inflammatory process through the release of cytokines and matrix metalloproteinases (MMPs), which can degrade the arterial extracellular matrix and promote further differentiation of macrophages into foam cells following uptake of oxLDL [42]. Cytokines from activated macrophages and endothelial cells result in the release of platelet-derived growth factor (PDGF), which, in turn, stimulates the migration of vascular SMCs from the media into the intima and support their proliferation [49,50]. Ultimately, foam cells derived from SMCs and macrophages die through both necrotic and apoptotic processes, thereby releasing their contents [51], and in this way, attract further macrophages. Secreted oxLDL molecules and dying foam cells accumulate in a necrotic core, a condensation site for further cellular debris of apoptotic macrophages and SMCs, which is surrounded by an endothelial layer and migrated SMCs. As the necrotic core progresses, calcium deposits further establish the atherosclerotic plaque that thins its fibrous cap along maturation and eventually becomes vulnerable to rupture [52]. When this luminal surface of the plaque is disrupted, the highly thrombogenic core is exposed, which ultimately leads to primary hemostasis. Locally impaired release of, e.g., the tissue factor pathway inhibitor, thrombomodulin or reduced expression of the endothelial protein C receptor on dysfunctional endothelial cells at the site of plaque rupture, further supports thrombus formation and prompts vessel stenosis, complete occlusion, and/or embolism [53,54,55,56,57]. 

A potential relationship between sphingolipids and atherosclerosis was first described by Smith in 1960 [58]. She reported that in the area of advanced lesions, human aortas present a higher proportion of lipids in the intima and media of the vessel wall. Specifically, sphingomyelin (SM) is increased in the intima of lesions sites compared to areas with less advanced lesions [58]. Sphingomyelin was found to account for 70–80% of all phospholipids in the necrotic core, indicating a potential pathophysiological role of sphingolipids in atherosclerosis—an observation that has been confirmed since then on several occasions [59,60,61,62]. Beyond SM, the presence of dihydroceramides (DhCer), ceramides (Cer), lactosylceramides (LacCer), glucosylceramides (GluCer), and sphingosine-1-phosphates (S1P) was subsequently identified as a common sphingolipid signature of carotid plaques [59]. In this review, we provide an overview of the disease modulating anti- and pro-atherogenic functions of each of these sphingolipids and discuss open aspects of the mechanistic pathophysiological relationship of these sphingolipids in the onset and progression of atherosclerosis. 

## 2. Dihydroceramide in Atherosclerosis Progression

### 2.1. Synthesis and Metabolism

**Figure 2:**
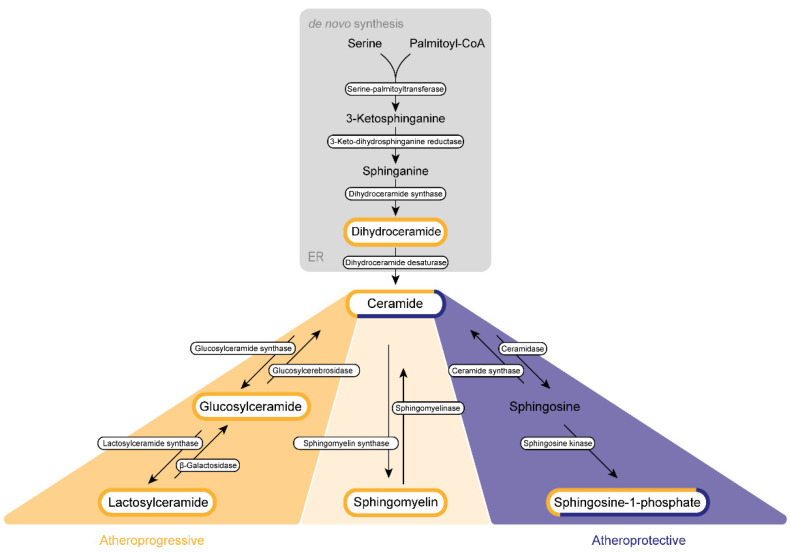
Sphingolipid biogenesis in atherosclerosis. Sphingolipids are synthesized de novo in the endoplasmic reticulum (ER) and the Golgi apparatus. Subsequently, they are transported via vesicles to the plasma membrane and the endosomes. The amino acid serine and palmitoyl-CoA provide the basis for the synthesis of 3-keto-sphinganine, which is reduced to sphinganine via 3-keto-dihydrosphinganine reductase. The dihydroceramide synthases form dihydroceramide, which can be catalyzed to ceramide, the backbone of all sphingolipids, by dihydroceramide desaturase. Ceramide itself can be converted into three further sphingolipid species. Glucosylceramide synthase mediates the production of glucosylceramide, which can be further modified to lactosylceramide through the enzyme lactosylceramide synthase. This modification can be reversed by β-galactosidase and glucosylcerebrosidase, respectively. Ceramide also provides the backbone for the generation of sphingomyelin via the activity of sphingomyelin synthase. Sphingosine-1-phosphate can be synthesized by ceramidase and sphingosine kinase. Several sphingolipids shown are assumed to exert influence on the progression of atherosclerosis. This impact can be categorized either as atherogenic (yellow) or as protective (purple) or can display characteristics of both categories (mixed).

The de novo synthesis of sphingolipid is initiated by a highly coordinated sequence of actions involving serine palmitoyltransferase, 3-keto-dihydrosphingosine reductase, and dihydroceramide synthase, which convert cytosolic serine and palmitoyl CoA molecules via sphinganine into DhCer (Figure 2).

DhCer is further processed at the endoplasmic reticulum (ER) membrane. Here, DhCer serves as a substrate for dihydroceramide desaturase that introduces a 4,5-trans-double bond to the sphingolipid backbone, thus generating Cer, which is further catalyzed by ceramidase and sphingosine kinases to first sphingosine and then S1P in the Golgi apparatus. Similar to most sphingolipids, DhCer is elevated in atherosclerotic plaques and is associated with inflammation and plaque instability [59].

### 2.2. Regulation of Inflammation

For a long time, no specific cellular function was attributed to DhCer, yet this notion has changed over the past 15 years, as DhCer was shown to impact autophagy, cell proliferation, cell survival and cell death in cancer and metabolic diseases [63,64,65,66,67]. In atheromatous plaques, DhCer levels positively correlate with proinflammatory cytokines such as monocyte chemoattractant protein-1, interleukin 6 (IL-6), and macrophage inflammatory protein-1 β. Over and above that, DhCer is able to induce the release of IL-6 in human coronary smooth muscle cells without inducing apoptosis [59]. However, caution is warranted in the interpretation of experimental results focusing on the specific function of DhCer, as pharmacological or genetic inhibition of enzymes involved in the de novo pathway will not only affect DhCer levels but also Cer concentration [68].

### 2.3. Regulation of Autophagy

In line with a potential functional role of DhCer in inflammatory processes per se, DhCer has been found to promote autophagy as demonstrated by the formation of autophagosomes in prostate cancer cells after stimulation with a DhCer desaturase inhibitor [69]. Of note, similar results were obtained by exogenous addition of short-chain DhCer [69]. Similarly, exogenous addition of DhCer analogues or treatment with DhCer desaturase inhibitors led to the accumulation of DhCer and promoted autophagy in cancer cells without causing cell death [64,65]. While a mechanistic link between DhCer and autophagy has thus been established, it remains a matter of controversy whether autophagy has a protective or a progressive effect on atherosclerosis. Normal autophagy flux is involved in vascular homeostasis, yet abnormal activity results in mechanisms aggravating atherosclerosis such as inducing thrombosis in endothelial cells, the secretion of pro-inflammatory cytokines by macrophages and abnormal remodeling of SMC in the intima. These characteristics can finally cause cell death and plaque instability [70]. Since short-chain DhCer can favor the formation of autophagosomes, it is appealing to hypothesize that short-chain DhCer also promotes autophagy in a pathophysiological context that may drive the progression of atherosclerosis. Moreover, the influence of DhCer on atherosclerosis promoting as well as atheroprotective mechanisms appears not to be restricted to autophagy only. DhCer has also been proposed to diminish apoptosis by inhibiting the formation of pores on the outer mitochondrial membrane, thereby impeding an essential step of the apoptotic cascade [71]. It remains to be evaluated whether and how this effect of DhCer on apoptosis influences atherosclerosis progression. In addition, DhCer affects oxidative stress by inducing ER stress. In contrast, DhCer levels are also elevated in the presence of oxidative stress, which can be explained by the inhibition of DhCer desaturase [72,73]. To investigate which effect provides the initiator for the other, further research is needed. 

## 3. Ceramide

The hydrophobic properties of ceramides restrict their solubility in an aqueous environment. Ceramides in plasma are therefore either bound to carrier proteins such as lipid transfer proteins or are associated with lipoproteins such as LDL and high-density lipoprotein (HDL). Cer provides the acyl-backbone for other sphingolipids such as S1P, GluCer, LacCer and SM. Besides the de novo pathway, the most physiologically relevant means of Cer synthesis is the acyl-CoA-dependent conversion of sphingosine and non-esterified fatty acids by the activity of a family of six ceramide synthases (CerS1-6) [74,75] into ceramides with distinct acyl chain lengths. Alternatively, ceramides can be metabolized by sphingomyelinases (SMases)-induced hydrolysis of sphingomyelin to Cer.

Importantly, Cer concentrations correlate with the risk for cardiovascular disease (CVD) in general and atherosclerosis specifically; as such, Cer qualifies as a prognostic marker for CVD as well as for sphingomyelin (SM) [76,77,78]. Since Cer is present in significantly enriched amounts in atherosclerotic plaques and has been shown to be correlated with aggregated [79] and circulating LDL [80], a causal relationship between Cer and atherosclerotic plaque progression has been assumed.

### 3.1. Sphingomyelinases (SMases)

It seems that an athero-promoting effect of Cer is mediated by specific types of SMases, e.g., Cer can be hydrolyzed from multiple SMases such as secreted lysosomal (L-SMase), acidic sphingomyelinase (A-SMase) and membrane neutral SMase (N-SMase). L-SMase and A-SMase are located in the endosome but can be translocated to the outer plasma membrane under certain conditions [81,82]. N-SMase, however, is synthesized predominantly in the ER and Golgi apparatus, but also in the inner leaflet of the plasma membrane. All three forms of SMase have been implicated in atheroprogression in distinct manners. High density lipoprotein (HDL) is one out of five major lipoproteins that transports lipid molecules within the body. HDL is usually referred to as “good cholesterol”, as it captures lipid molecules in the artery walls and thereby prevents atheroprogression [83,84]. HDL molecules mainly consist of apolipoprotein A (ApoA) and further apolipoprotein C (ApoC). The main function of ApoC-1 protein is the inhibition of cholesterol ester transfer protein (CETP) and inhibiting the lipoprotein binding to the “bad cholesterols” high density lipoprotein (HDL) and very low density lipoprotein (VLDL). Mutations reducing the function of CETP have thereby been associated with elevated atherosclerosis progression [85]. This pathophysiological mechanism seems to be of crucial role in terms of the involvement of Cer in atherogenesis, since ApoC-1-enriched HDL induces apoptosis and cell death of vascular smooth muscle cells (VSMC) via N-SMase activation [86]. Furthermore, oxLDL induces proliferation of VSMC via N-SMase [87,88]. Since both apoptosis and proliferation of VSMC are mechanisms associated with atherogenesis, these findings may suggest an atheroprogressive effect of N-SMase activation. Similar pro-atherogenic effects have been described for A-SMase. Endothelial cells secrete A-SMase, which hydrolyses SM on the surface of atherogenic lipoproteins to Cer and thus mediates the fusion, aggregation and affinity of lipoprotein particles with/at/toward the endothelium of arteries [89]. Analyses of *ApoE*^-/-^/*Ldlr*^-/-^/*Smpd1*^-/-^ triple knockout mice highlighted the impact of A-SMase on atherogenesis, since the absence of A-SMase reduced the formation of atherosclerotic lesions and arterial trapping of atherogenic lipoproteins in the otherwise atheroprone *ApoE*^-/-^/*Ldlr*^-/-^ mice [90]. Similar to A-SMase, L-SMase has also been found to promote the pathogenesis of atherosclerosis. As a result of ligand binding to TNF receptors, activation and translocation of L-SMase proceeds. Grassme et al. identified a mechanism by which L-SMase seems to enhance atherosclerosis [91] through the formation of Cer-enriched domains. These domains are formed by receptor-mediated translocation of L-SMase. L-SMase is primarily localized in the endolysosomal compartment and can be relocated to the outer leaflet of the plasma membrane upon stimulation via CD95 receptor [92,93,94]. Due to this translocation, sphingolipid-rich domains accumulate and release extracellularly orientated Cer. Accumulation of Cer leads to the formation of Cer-enriched platforms on the surface, which, in turn, efficiently initiate apoptosis signaling by trapping and clustering the receptors. The aggravating effect on atherosclerosis is postulated since the Cer-enriched membrane domains in VSMC and EC impair the vasodilatory properties in ECs and VSMC [95,96] and enhance muscarinic-1 receptor-mediated constriction of coronary arteries [97]. 

Overall, the three types of SMases have been implicated at several levels in the progression of atherosclerosis. However, as these studies have been typically performed in different models without back-to-back comparisons of the role of different SMases, it remains to be shown whether the individual roles of L-SMase vs. A-SMase or N-SMase in atherogenesis are specific or redundant.

### 3.2. Regulation by Matrix Metalloproteinases (MMPs)

Activation of the oxLDL-induced SM/Cer pathway and subsequent activation of ERK1/2 is regulated by MMPs, a large family of zinc proteases [98]. In principle, MMP content is increased in atheromatous plaques and has been associated with plaque instability and the formation of stenotic lesions that recur after treatment [99]. The expression of these MMPs is regulated and activated by major triggers of vascular remodeling such as inflammation or oxidative stress [100]. In SMC, the connection between MMPs and atherogenesis is considered to be mediated by oxLDL-induced activation of N-SMase, in that inhibition of MMP-2 inhibits N-SMase and as such, Cer production. Conversely, exogenous MMP-2 activates the SM/Cer pathway, supporting the notion of an oxLDL-induced activation of the Cer pathway via activation of N-SMase [98]. However, the exact mechanism by which oxLDL activates SMases via MMPs is currently unclear and remains the scope for future research. These findings highlight, thus far, the atheroprogressive functions of Cer and related mediators as SMases and MMPs. As we will discuss in the next paragraph, inflammatory mediators may exert an additional influence on the effects of sphingolipids on cellular mechanisms such as apoptosis or vasodilation.

### 3.3. Regulation by Tumor Necrosis Factor Alpha (TNFα)

Tumor necrosis factor alpha (TNFα) is likely a central factor that further increases Cer concentrations in atherosclerotic lesions [101,102]. TNFα contributes to endothelial dysfunction by stimulating ROS production and induces the expression of various inflammatory cytokines and chemokines [103,104,105,106]. Acting on the vascular endothelium, TNFα thus emerges as a key driver for the progression of atherosclerosis. Linking TNFα to sphingolipids, Sawada et al. proposed a TNFα-induced increase in Cer levels in human glioma cells via two different pathways, both of which are initiated by activation of caspase-8: first, a p53 and ROS-dependent pathway that leads to N-SMase activation via GSH depletion and thus to increased production of Cer; a second pathway activates A-SMase directly via caspase-8, and, thus, causes a ROS-independent increase in Cer levels resulting in a TNFα-induced apoptosis of human glioma cells [107]. Analogously, clinical studies have shown that the ischemic myocardium is stimulated by inflammatory cytokines such as TNFα, interleukin 2 and endostatin, similarly resulting in an A-SMase- and N-SMase-dependent elevation of Cer levels [108,109]. However, the effect of TNFα on Cer production is not unidirectional. TNFα can also be induced by stimulating human umbilical vein endothelial cells with C2-Cer [110]. It may thus be inferred that TNFα not only stimulates Cer production, but conversely, Cer synthesis also stimulates TNFα release—thus establishing a pathological feedback loop. This notion is in line with studies showing that anti-TNFα therapy is able to improve endothelial function in humans with vascular inflammation [111,112]. Nevertheless, it remains to be shown whether anti-TNFα treatment may reduce vascular ceramide production and attenuate CVD and atherosclerosis. Of interest, changes in amino acid metabolism may also affect Cer de novo synthesis, as homocysteine leads to increased formation of superoxide anions by stimulation of the NADPH oxidase pathway [113]. In agreement with this hypothesis, ceramide levels increase in response to rising homocysteine concentrations via the de novo synthesis pathway rather than the SMase pathway, as treatment with myriocin (a highly selective serine palmitoyltransferase inhibitor) reduced homocysteine-induced ceramide production in rats [114]. In summary, ceramide is produced by two independent synthesis pathways: (i) SMase-dependent hydrolysis from sphingomyelin and (ii) de novo synthesis via ceramide synthase, both of which are assumed to be stimulated in atherosclerosis in general and by inflammatory cytokines such as TNFα specifically. 

In conclusion, Cer has been demonstrated to be detrimental in atherosclerosis as (i) being enriched in atherosclerotic plaques, (ii) SMases being involved in formation of aortic lesions and processes involved in atherogenesis such as apoptosis or lipoprotein trapping and (iii) Cer levels being elevated in response to MMPs and TNFα—which are also elevated in atherosclerotic lesions—via SMase activation. The underlying mechanisms of action are probably diverse, only partially elucidated, and will be discussed in the following sections.

### 3.4. Regulation of NO Production

Under physiological conditions, vascular NO production is stimulated by shear stress, catalyzed by the endothelial NO synthase (eNOS), and constitutes an essential feature of endothelial cell function and vascular homeostasis. Reduced NO release or impaired NO bioavailability are key factors in the progression of endothelial dysfunction, manifested by loss of endothelium-dependent vasorelaxation. Cer is considered an important negative regulator of endothelial NO production, as it decreases the release of NO from human umbilical vein endothelial cells [115,116] and initiates the production of superoxide anions [117,118,119]. As such, Cer may promote endothelial dysfunction by decreasing NO and increasing ROS production, and thus promote the development of atherosclerosis.

### 3.5. Regulation of LDL Aggregation

Another essential role of ceramide in the development of atherosclerosis is the ceramide-induced aggregation of LDL. Increased levels of Cer correlate with the ability of LDL to form aggregates [120,121,122,123]. During atherogenesis, LDL is enriched at the vessel membrane where it is exposed to SMase. OxLDL activates SMase to convert LDL-SM to Cer within atherosclerotic lesions [75,122]. Cer, in turn, enables a conformational change in apolipoprotein B100 (ApoB100), which provides the essential step for LDL molecules to aggregate [124,125,126]. This process is further accompanied by macrophage-mediated phagocytosis and foam cell formation, aggravating atherosclerotic lesion formation [92,127,128]. In line with this concept, the use of the sphingolipid synthesis inhibitor myriocin prevents aggregation of LDL and succeeds in a reduction of plaque formation [120,127].

In addition to its ability to promote oxidative stress and to enhance LDL aggregation, Cer causes apoptosis and necrosis in human coronary artery smooth muscle cells in vitro [59], which further accentuate its pro-atherosclerotic function.

More recent findings have also taken into account a more differentiated view on the distinct role of certain molecular species of ceramide. Long chain (C11–C20), very long chain (C21–C24) and ultra-long chain (>C24) ceramide species are formed in the sphingolipid synthesis pathway by six different Cer synthases (CerS1-6) with specific affinities for the chain length of the fatty acyl-CoA. Deletion or pharmacologic inhibition on N-SMase2 in the ApoE^-/-^ mouse model reduced atherosclerotic lesions and decreased macrophage infiltration and lipid deposition via small interfering RNAs in the nuclear factor erythroid 2-related factor 2 pathway [129]. This species-dependent effect on the biological activities of Cer was underscored by overexpression of CerS4 and CerS6, which generate long chain Cer to inhibit cell proliferation while inducing apoptosis, respectively. CerS2, in turn, forms very long chain Cer that increases cell proliferation [128,130]. This highlights the importance of the activity of specific CerS and subsequent changes in Cer species composition in the initiation and progression of atherosclerosis and remains a point of consideration in the understanding of the pathophysiology of CVD.

## 4. Sphingosine-1-Phosphate

The cleavage of fatty acids from the sphingolipid backbone of Cer by ceramidases releases sphingosine, which can be further phosphorylated by the activation of sphingosine kinase isoenzymes 1 and 2 (Sphk1, Sphk2) to spingosine-1-phosphate [131]. Sphk1 and Sphk2 are highly conserved and present in most mammalian cells and tissues, including platelets [132], erythrocytes [133], and the endothelium itself [134] which secrete S1P by the specific S1P-transporters major facilitator superfamily domain containing 2B (MFSD2B, erythrocytes and platelets) and spinster-homologue-2 (SPNS2, endothelial cells) into plasma and lymph [135,136,137,138]. Here, S1P signals as a bioactive lipid mediator by targeting five different G protein-coupled S1P-receptors (S1PR1-5) on various hematopoietic and vascular cells, and thereby controls cellular proliferation, apoptosis and cell migration in the blood vasculature and interstitial spaces and regulates endothelial barrier function [139]. Therefore, S1P/S1PR signaling may infer a significant role in the pathogenesis of atherosclerotic cardiovascular disease. Serum S1P is a strong and robust predictor of the occurrence of obstructive coronary artery disease [140], suggesting a correlation with atherogenic effects. Furthermore, the S1PR modulator FTY720, which acts upon all S1PRs except S1PR2 [141], effectively attenuates atherogenesis in ApoE- and LDL-receptor (LDL-R) deficient mice, respectively [142,143], implicating an atheroprotective effect. Future research should further confirm these contradictory initial findings.

To realize signaling in health and disease, S1P has to bind to chaperone proteins including apolipoprotein M (ApoM) on HDL (~65% of all free plasma S1P) or albumin (~30% of all free plasma S1P) and LDL or VLDL (<5% of all plasma S1P), as its hydrophobic backbone and polar phosphate head group restrict the membrane permeability of S1P [144,145,146]. The plasma S1P levels also closely correlate to levels of total cholesterol, LDL cholesterol and HDL cholesterol in normolipidemic healthy subjects [147,148]. These associations may be of mutual functional relevance, e.g., the interaction of S1P and HDL has been proposed to reinforce their anti-thrombotic, anti-inflammatory and antioxidant properties [149]. The S1P/cholesterol interrelation has been experimentally validated by gain-of-function mutations of the LDL-R in livers of mice, which reduced S1P and ApoM levels in wildtype but not in ApoE-deficient mice. This finding suggests ApoE-dependent clearance of ApoM-associated S1P [150]. In line with this notion, statin treatment reduced serum ApoM levels in type 2 diabetes mellitus patients [151]. Further, only the S1P/ApoM complex on HDL is able to activate endothelial S1PR_1_ Gi-signaling and downstream ERK- and Akt-signaling, preserving endothelial adherent junctions [145] and decreasing TNFα-induced activation of nuclear factor kappa B (NFκB) and expression of ICAM-1 [152], while this endothelium-protective signaling cascade is only insufficiently activated by the S1P/albumin complex [153]. 

The majority of receptor-associated actions of S1PR-mediated intracellular processes are atheroprotective. Evidence from in vivo experiments shows that S1PR_1_ and S1PR_3_ are essential for both maintenance of endothelial barrier function, as the receptors’ downstream signaling cascade stabilizes endothelial cell–cell junctions [154] and attenuates endothelial contraction [155], and vascular relaxation by phosphorylation of eNOS and subsequently increased endothelial NO release [156,157,158]. In addition, a protective function against the development of atherosclerotic lesions has been suggested, as expression of the adhesion molecules VCAM-1 and ICAM-1 can be inhibited by S1PR_1_ signaling, thus reducing leukocyte adhesion and subsequent extravasation [157,158]. Analogously, S1P signaling via S1PR_3_ can inhibit the recruitment of inflammatory neutrophils and suppress apoptosis of cardiomyocytes. S1PR_3_-deficient mice are accordingly more susceptible for infarction in a mouse model of myocardial ischemia/reperfusion as compared to their corresponding wild type [159]. In contrast to this anti-inflammatory role of S1PR_3_ signaling, however, S1PR_3_ deficiency in ApoE^-/-^ mice was found to strongly reduce monocyte recruitment by decreasing monocyte chemoattractant protein-1 secretion without affecting the size of atherogenic lesions [160]. These pro-inflammatory and, hence, potentially atherogenic properties of S1P signaling are further supported by the finding that S1PR_1_ enhances chemotaxis of lymphocytes and natural killer cells (NK) and, thus, has pro-inflammatory and pro-atherosclerotic properties [153]. S1P signaling through S1PR_2_ is even more likely to be associated with atherogenic functions. Although S1PR_2_ has been shown to inhibit SMC migration [161], it is centrally involved in the recruitment of inflammatory macrophages [162]. As such, S1PR_2_^-/-^/ApoE^-/-^-double-deficient mice show reduced release of IL-18 and IL-1β, leading to impaired interstitial macrophage recruitment and, consequently, reduced formation of atherosclerotic plaques and necrotic cores in comparison to S1PR_2_-proficient mice [163]. Consistently, S1PR_2_-deficient macrophages express less CD36 and scavenger receptors ex vivo and increase cholesterol efflux while decreasing oxLDL uptake [163]. Atherogenic effects of S1PR_2_ signaling have also been suggested based on the fact that S1P can impair endothelial barrier function via the S1PR2/Rho/ROCK pathway [164]. However, S1PR_2_ deficiency in mice is associated with an increased risk of seizures and the development of B-cell lymphomas, arguing against the suitability of this receptor as a therapeutic target in atherosclerosis [165,166,167]. S1PR_4_, expressed on leukocytes, NK cells and airway SMC [168], and S1PR_5_ expressed on NK cells and oligodendrocytes [169] have not been associated with atheroprogression to date, even though S1PR_4_ stimulates IL-10 secretion from T-cells and simultaneously inhibits interleukin 4 and interferon-γ production [170], while S1PR_5_ mobilizes NK cells during infections. Although these findings may suggest an indirect involvement of S1PR_4_ and S1PR_5_ in atherosclerosis-associated inflammation, the latter receptors seem to have less of a direct impact on atherosclerosis pathology.

## 5. Sphingomyelin (SM)

SM is the most abundant sphingolipid in mammalian tissues, where it serves as an important structural component of cell and plasma membranes [171]. Importantly, in the context of atherosclerosis, SM is also involved in maintaining cholesterol homoeostasis, as addition of exogenous SM to cells increases cholesterol biosynthesis and affects LDL binding to cell surface receptors [172,173]. However, there is further evidence implicating SM in the pathogenesis of atherosclerosis. SM has been identified as one component of human atherosclerotic plaques, and its abundance correlates with histological markers of plaque instability and is associated with the expression of pro-inflammatory cytokines. In accordance with this observation, stimulation of human coronary smooth muscle cells with SM in vitro induces a pro-inflammatory response reflected by IL-6 release [59]. SM plasma levels of atherosclerotic ApoE^-/-^ mice are also elevated in comparison to WT mice [174]. Likewise, rabbits with hypercholesterolemia show elevated levels of SM compared with other lipids in atherosclerotic lesions [175]. Similar to S1P, SM in plasma is associated with VLDL/HDL cholesterol (63–75%) and LDL cholesterol (25–35%). The emerging notion that elevated SM levels in plasma are associated with pro-atherogenic properties is further supported by the fact that a decrease in HDL SM content is associated with smaller and more dense HDL. These complex lipoprotein particles favor cholesterol efflux, anti-oxidative activity toward LDL oxidation, antithrombotic activity in human platelets, as well as anti-inflammatory and anti-apoptotic activity [176]. In accordance, anti-apoptotic and anti-oxidative activities of small compact HDL cholesterol have been associated with SM degradation [177]. 

Unlike SMase, which hydrolyzes SM to Cer, the sphingomyelin synthase (SMS) catalyzes the synthesis of SM from Cer. SMS represents a family of different isoforms: SMS1 is primarily localized in the Golgi apparatus, whereas SMS2 primarily in plasma membranes [178,179].

Inhibition of SMS1 has been proposed as a potential therapeutic approach in atherosclerosis, as SMS1^-/-^ mice show a decreased atherosclerotic phenotype characterized by reduced atherosclerotic lesions in the entire aortas as well as decreased macrophage content in these lesions [180]. Similar effects have been achieved in SMS2-deficient mice. These mice are marked by a reduction in secretion of pro-inflammatory cytokines, which is accompanied by the reduction of atherosclerotic lesions, necrotic core formation, macrophage content and collagen content compared to wild-type mice [181]. The pro-atherogenic capabilities of SM are further confirmed, as adenovirus-mediated insertion of SMS2 in ApoE ^-/-^ mice results in an increase in atherosclerotic lesions [182]. Similarly, SMS2 is shown to act as a modulator of NF-κB activation in HEK193 cells and macrophages from SMS2-deficent mice. This could provide one mechanistic explanation of the pro-atherogenic function of SM [183]. Consistent with this pro-atherogenic character of SM, overexpression of SMS1 and SMS2 increases the lipoprotein atherogenic potential in mice [184], whereas the simultaneous deficiency of SMS1 and SMS2 leads to a reduction in plasma SM and pro-inflammatory cytokine secretion [180]. In this context, it is remarkable that the inhibition of SMS1 alone leads to a decrease in the SM content in plasma, but simultaneously to an increase in DhCer and Cer in the plasma. Considering those two being associated with both atheroprotective and atherogenic effects, an explicit categorization of SM as an atheroprotective should only be made with caution. Further, it will be crucial to determine the mechanistic interplay between the inhibition of SMS1 and the increase in DhCer and Cer in order to identify a definite therapeutic signaling cascade. With regard to the identification of potential novel therapeutic targets, it is furthermore relevant to consider that loss-of-function by deletion of SMS1 (similar to S1PR2, vide supra) entails serious side effects such as low-frequency hearing loss [179,185], impaired insulin secretion [186], or CD4+ cell dysfunction [187].

## 6. LacCer and GluCer—Sphingolipids with Non-Chimeric Functions?

Lactosylceramide synthase (LacCerS) generates LacCer by transferring galactose from uridine diphosphate-galactose to GluCer. LacCer and GluCer are classified as glycosphingolipids whose synthesis can be inhibited by D-threo-1-phenyl-2-decanoylamino-3-morpholino-1-propanol (D-PDMP). D-PDMP is an analog of glucosylceramide originally synthesized to inhibit glcosylceramide synthase in patients with Gaucher’s disease [188,189]. However, D-PDMP has also been shown to be directly capable of LacCerS [188]. These inhibitory effects of D-PDMP of LacCer and GluCer synthases have been utilized to identify the involvement of these sphingolipids in terms of atherogenesis. Thereby, it was shown that LacCer and GluCer increase vascular dysfunction, since aortic wall thickening, presence of Ca^2+^ deposits and vascular stiffness were decreased upon blockade of glycosphingolipid synthesis in ApoE^-/-^ mice [189].

This finding is strengthened by previous studies showing that LacCer exerts an influence on many mechanisms relevant to atherosclerosis. For example, LacCer mediates TNF-induced NF-κB expression and ICAM-1 expression in endothelial cells by activation of a redox-dependent transcriptional pathway [190,191]. In the same manner, it has atheroprogressive effects by stimulating the expression of MAC1 on monocytes or neutrophils, presumably facilitating their adhesion to endothelial cells and initiating atherosclerosis [189]. Based on the results of various studies, Chatterjee and colleagues postulated the following pathway to mechanistically explain LacCer-induced atherosclerosis progression: OxLDL increases the production of endogenous LacCer, which, in turn, activates NADPH oxidase [118,191,192,193]. The resulting production of superoxide [190,194,195] induces GTP loading of P21ras and thus activation of a kinase cascade from Raf-2, Mek2 and p44MAPK. Phosphorylation of p44MAPK results in a local shift of p44MAPK from the cytoplasm to the nucleus [118,191]. This step determines the final expression of c-fos, proliferating nuclear antigen and cell proliferation. 

GluCer has previously been implicated in arterial stiffness and vascular cell wall thickening [189] but, in addition, appears to have a direct impact on atherosclerotic plaque development, as inhibition of glucosylceramide synthase attenuates atherosclerotic plaque development and the expression of inflammatory genes [196]. These glycosylceramide synthase-associated effects were found even more pronounced in ApoE*leiden mice, in which pharmacological inhibition of glucosylceramide synthase also led to a drastic reduction of atherosclerotic plaques. This effect was accompanied by a decreased cholesterol level in the liver and an increased excretion of cholesterol by feces and an increased secretion of bile [196]. The effects could also be replicated in LDL receptor KO mice. In vitro, glucosylceramide per se initiates apoptosis in HCASMC and induces an inflammatory response, evident as increased expression of IL-6, MCP- and macrophage inflammatory protein-1β [59]. In view of these reported functions, LacCer and GluCer seem to exhibit primarily pro-atherogenic effects.

## 7. Conclusions

The large quantity of sphingolipids identified in atherosclerotic plaques supports a possible link between sphingolipids and atherosclerosis. The key question, however, that remains to be clarified is whether sphingolipids are the cause or the consequence of atherogenesis. Various studies have demonstrated a specific effect of sphingolipids on cellular processes relevant to the development of atherosclerosis, such as impaired NO production [109,115,116,117,118,119], apoptosis [59,71,86,107], plaque development [70,79,80,120,127,196], or LDL aggregation [59,80,125]. As a function of their cellular and tissue context or their respective sphingoid bases, however, the sphingolipids Cer, DhCer, GluCer, LacCer, SM and S1P can exert chimeric and often opposing functions in the pathogenesis and progression of atherosclerosis (Table 1). While the existing evidence reviewed herein suggests that SM, DhCer, LacCer and GluCer exclusively mediate atheroprogressive effects, Cer and S1P may exert both protective as well as progressive properties in atherosclerosis (Table 1). As such, S1P mediates anti-apoptotic [139,159] and anti-inflammatory [162,170] processes as well as enhancing vasoconstriction [149,155] while maintaining endothelial barrier function [145,154] (Table 1). In contrast, its pro-atherogenic functions are evident in its ability to activate lymphocytes [153] and to promote primary hemostasis and thrombus formation [163] (Table 1). Furthermore, it should be considered that the synthetic pathway of sphingolipids is intertwined, and enzymes that can synthesize multiple species of a sphingolipid class with unique properties mediate the generation of one from another. Very long chain Cer is pro-thrombotic, induces cell proliferation and TNFα secretion, and correlates with LDL aggregation, whereas long chain Cer inhibits proliferation and induces apoptosis [128,130]—highlighting the chimeric properties of different Cer species depending on their sphingoid bases (Table 1). This opposing mode of action within the same class of sphingolipids is influenced by their biosynthesis, as for, e.g., Cer, the activity of CerS1-6 results in the generation of Cer with a distinct chain length, which has unique progressive or protective functions on atherogenesis [128,130]. Furthermore, sphingolipid receptors can essentially determine whether the mediated effect is atheroprotective or atherogenic, as exemplified by the differential expression and upstream signaling facilitated by S1PR1-5 [139,141,142,143]. In addition, an increasingly recognized level of regulation is the bioavailability of S1P mediated by its specific carrier molecules. While ApoM-associated S1P mediates atheroprotective effects, S1P bound to albumin can mediate either atheroprotective or atheroprogressive effects [145,150,152].

In a broader context, the functions of sphingolipids are determined by such a number of individual factors and steps such that one may wonder about the evolutionary purpose of this complexity. This could serve as an amplification process, so that many individual steeps enhance the effect, explaining the involvement of many cell types and molecules in the context of atherosclerosis. Furthermore, this signaling network might reflect a system of mutual checks and balances, ensuring that not a single imbalance leads immediately to the formation of atherosclerosis, thus preventing atherosclerosis from developing rapidly. A deeper understanding of the complex sphingolipid network and the chimeric properties of individual sphingolipid classes and species offers new therapeutic possibilities. For example, knowledge of the biosynthesis of different species of a sphingolipid, each with chimeric functions, opens up therapeutic strategies that allow for targeted inhibition of enzymes that lead to the formation of atherosclerosis-promoting sphingolipids and, consequently, could maximize the therapeutic outcome. Taken together, this may imply that sphingolipids and their actions should be analyzed as a network rather than as individual components.

## Figures and Tables

**Table 1:** Sphingolipids and their associated mechanism in atherogenesis. Sphingolipids exhibit molecular mechanisms, which are either categorized as atheroprotective or atheroprogressive.

Sphingolipid	Associated Mechanism	Effect on Atherosclerosis	References
Dihydroceramide	↑ Autophagy ↑ Oxidative stress ↑ Inflammatory cytokines ↑ Cell proliferation ↑ Plaque instability	Progressive	[59,63,64,65,66,67,69,71,72,73]
Cer Long-chain Very long-chain	↑ Inflammation ↑ Proliferation ↑ LDL-Aggregation ↓ Cell proliferation ↑ Apoptosis ↑ Cell proliferation	Progressive Protective Progressive	[28,29,30,32,59,76,90,100,101,102,128,130]
S1P S1PR_1_ S1PR_2_ S1PR_3_ S1P/ApoM S1P/Albumin	↑ Endothelial barrier function ↓ Apoptosis ↑ Chemotaxis of lymphocytes and NK cells ↓ ICAM1 and VCAM1 expression ↓ Barrier function ↑ Recruitment of inflam. macrophages ↑ Plaque and necrotic core formation ↓ SMC migration ↑ Endothelial barrier function ↑ Monocyte recruitment ↓ Thrombus formation ↓ Inflammation ↓ Apoptosis Not shown	Protective Progressive Progressive Protective Progressive Protective Protective Progressive Protective Protective + progressive	[59,139,141,143,145,149,150,152,153,156,157,158,160,161,162,163,164]
Sphingomyelin	↑ Hypercholesterolemia ↑ Apoptosis ↑ Inflammatory cytokines ↑ Thrombus formation ↑ Plaque instability ↑ Atherosclerotic lesions ↑ Macrophage content in lesions	Progressive	[59,174,175,176,177,180,181,182,183,184]
Lactosylceramide	↑ TNFα-induced NFκB expression ↑ ICAM-1 expression ↑ MAC1 expression ↑ Arterial stiffness ↑ Aortic wall thickening ↑ Presence of aortic Ca^2+^ deposits ↑ Apoptosis ↑ Inflammatory cytokines	Progressive	[59,118,189,190,191,192,193,197]
Glucosylceramide	↑ Arterial stiffness ↑ Aortic wall thickening ↑ Presence of aortic Ca^2+^ deposits ↑ plaque development ↑ cholesterol level liver ↑ Apoptosis ↑ Inflammatory cytokines	Progressive	[1,2,5]

## References

[1] Thudichum J. (1884). A Treatise on the Chemical Constitution of the Brain Bailliere.

[2] Murray C.J., Lopez A.D. (1997). Mortality by cause for eight regions of the world: Global Burden of Disease Study. Lancet.

[3] Kelly B.B., Fuster V. (2010). Promoting Cardiovascular Health in the Developing World: A Critical Challenge to Achieve Global Health.

[4] Badimon L., Vilahur G. (2014). Thrombosis formation on atherosclerotic lesions and plaque rupture. J. Intern. Med..

[5] Zhang S.H., Reddick R.L., Burkey B., Maeda N. (1994). Diet-induced atherosclerosis in mice heterozygous and homozygous for apolipoprotein E gene disruption. J. Clin. Investig..

[6] Reddick R.L., Zhang S.H., Maeda N. (1994). Atherosclerosis in mice lacking apo E. Evaluation of lesional development and progression. Arter. Thromb..

[7] Dutta P., Courties G., Wei Y., Leuschner F., Gorbatov R., Robbins C.S., Iwamoto Y., Thompson B., Carlson A.L., Heidt T. (2012). Myocardial infarction accelerates atherosclerosis. Nature.

[8] Palasubramaniam J., Wang X., Peter K. (2019). Myocardial Infarction-From Atherosclerosis to Thrombosis. Arter. Thromb. Vasc. Biol..

[9] Shi Y., Guo L., Chen Y., Xie Q., Yan Z., Liu Y., Kang J., Li S. (2021). Risk factors for ischemic stroke: Differences between cerebral small vessel and large artery atherosclerosis aetiologies. Folia Neuropathol..

[10] Libby P., Ridker P.M., Maseri A. (2002). Inflammation and atherosclerosis. Circulation.

[11] van Varik B.J., Rennenberg R.J., Reutelingsperger C.P., Kroon A.A., de Leeuw P.W., Schurgers L.J. (2012). Mechanisms of arterial remodeling: Lessons from genetic diseases. Front. Genet..

[12] Rafieian-Kopaei M., Setorki M., Doudi M., Baradaran A., Nasri H. (2014). Atherosclerosis: Process, indicators, risk factors and new hopes. Int. J. Prev. Med..

[13] Aronson D., Rayfield E.J. (2002). How hyperglycemia promotes atherosclerosis: Molecular mechanisms. Cardiovasc. Diabetol..

[14] Glasser S.P., Selwyn A.P., Ganz P. (1996). Atherosclerosis: Risk factors and the vascular endothelium. Am. Heart J..

[15] Landmesser U., Hornig B., Drexler H. (2004). Endothelial function: A critical determinant in atherosclerosis?. Circulation.

[16] Muller G., Goettsch C., Morawietz H. (2007). Oxidative stress and endothelial dysfunction. Haemostaseologie.

[17] Gryglewski R.J., Palmer R.M., Moncada S. (1986). Superoxide anion is involved in the breakdown of endothelium-derived vascular relaxing factor. Nature.

[18] Zou M.H., Cohen R., Ullrich V. (2004). Peroxynitrite and vascular endothelial dysfunction in diabetes mellitus. Endothelium.

[19] Vergnani L., Hatrik S., Ricci F., Passaro A., Manzoli N., Zuliani G., Brovkovych V., Fellin R., Malinski T. (2000). Effect of native and oxidized low-density lipoprotein on endothelial nitric oxide and superoxide production: Key role of L-arginine availability. Circulation.

[20] Rueckschloss U., Galle J., Holtz J., Zerkowski H.R., Morawietz H. (2001). Induction of NAD(P)H oxidase by oxidized low-density lipoprotein in human endothelial cells: Antioxidative potential of hydroxymethylglutaryl coenzyme A reductase inhibitor therapy. Circulation.

[21] Görlach A., Brandes R.P., Nguyen K., Amidi M., Dehghani F., Busse R. (2000). A gp91phox containing NADPH oxidase selectively expressed in endothelial cells is a major source of oxygen radical generation in the arterial wall. Circ. Res..

[22] Jones S.A., O’Donnell V.B., Wood J.D., Broughton J.P., Hughes E.J., Jones O.T. (1996). Expression of phagocyte NADPH oxidase components in human endothelial cells. Am. J. Physiol..

[23] Lüscher T.F., Barton M. (1997). Biology of the endothelium. Clin. Cardiol..

[24] Stocker R., Keaney J.F. (2004). Role of oxidative modifications in atherosclerosis. Physiol. Rev..

[25] Pennathur S., Heinecke J.W. (2007). Oxidative stress and endothelial dysfunction in vascular disease. Curr. Diab. Rep..

[26] Mundi S., Massaro M., Scoditti E., Carluccio M.A., Van Hinsbergh V.W., Iruela-Arispe M.L., De Caterina R. (2018). Endothelial permeability, LDL deposition, and cardiovascular risk factors—A review. Cardiovasc. Res..

[27] Stemerman M.B. (1981). Effects of moderate hypercholesterolemia on rabbit endothelium. Arterioscler. Off. J. Am. Heart Assoc. Inc..

[28] De Caterina R., Libby P. (2008). Endothelial Dysfunctions in Vascular Disease. https://books.google.co.jp/books?hl=zh-TW&lr=&id=et-Pl-xh2vQC&oi=fnd&pg=PP2&dq=Endothelial+Dysfunctions+in+Vascular+Disease&ots=C5lFSmT374&sig=-OnsuY2im7CVRTVSg8WeMkMUgV4&redir_esc=y#v=onepage&q=Endothelial%20Dysfunctions%20in%20Vascular%20Disease&f=false.

[29] Nording H., Baron L., Langer H.F. (2020). Platelets as therapeutic targets to prevent atherosclerosis. Atherosclerosis.

[30] von Hundelshausen P., Weber K.S., Huo Y., Proudfoot A.E., Nelson P.J., Ley K., Weber C. (2001). RANTES deposition by platelets triggers monocyte arrest on inflamed and atherosclerotic endothelium. Circulation.

[31] OYu P., Peclo M., Gown A. (1992). Various cell types in human atherosclerotic lesions express ICAM-1. Further immunocytochemical and immunochemical studies employing monoclonal antibody 10F3. Am. J. Pathol..

[32] Poston R., Haskard D., Coucher J., Gall N., Johnson-Tidey R. (1992). Expression of intercellular adhesion molecule-1 in atherosclerotic plaques. Am. J. Pathol..

[33] Krieglstein C.F., Granger D.N. (2001). Adhesion molecules and their role in vascular disease. Am. J. Hypertens..

[34] Navab M., Imes S., Hama S., Hough G., Ross L., Bork R., Valente A., Berliner J., Drinkwater D., Laks H. (1991). Monocyte transmigration induced by modification of low density lipoprotein in cocultures of human aortic wall cells is due to induction of monocyte chemotactic protein 1 synthesis and is abolished by high density lipoprotein. J. Clin. Investig..

[35] Pham-Huy L.A., He H., Pham-Huy C. (2008). Free radicals, antioxidants in disease and health. Int. J. Biomed. Sci. IJBS.

[36] Wolff S.P., Garner A., Dean R.T. (1986). Free radicals, lipids and protein degradation. Trends Biochem. Sci..

[37] Hurt-Camejo E., Camejo G., Rosengren B., Lopez F., Ahlström C., Fager G., Bondjers G. (1992). Effect of arterial proteoglycans and glycosaminoglycans on low density lipoprotein oxidation and its uptake by human macrophages and arterial smooth muscle cells. Arterioscler. Thromb. A J. Vasc. Biol..

[38] Zhu S.N., Chen M., Jongstra-Bilen J., Cybulsky M.I. (2009). GM-CSF regulates intimal cell proliferation in nascent atherosclerotic lesions. J. Exp. Med..

[39] Subramanian M., Thorp E., Tabas I. (2015). Identification of a non-growth factor role for GM-CSF in advanced atherosclerosis: Promotion of macrophage apoptosis and plaque necrosis through IL-23 signaling. Circ. Res..

[40] Gordon S., Martinez F.O. (2010). Alternative activation of macrophages: Mechanism and functions. Immunity.

[41] Williams H.J., Fisher E.A., Greaves D.R. (2012). Macrophage differentiation and function in atherosclerosis: Opportunities for therapeutic intervention?. J. Innate Immun..

[42] Steinberg D. (1997). Low density lipoprotein oxidation and its pathobiological significance. J. Biol. Chem..

[43] Febbraio M., Podrez E.A., Smith J.D., Hajjar D.P., Hazen S.L., Hoff H.F., Sharma K., Silverstein R.L. (2000). Targeted disruption of the class B scavenger receptor CD36 protects against atherosclerotic lesion development in mice. J. Clin. Investig..

[44] Ricciarelli R., Zingg J.-M., Azzi A. (2000). Vitamin E reduces the uptake of oxidized LDL by inhibiting CD36 scavenger receptor expression in cultured aortic smooth muscle cells. Circulation.

[45] Aoyama T., Chen M., Fujiwara H., Masaki T., Sawamura T. (2000). LOX-1 mediates lysophosphatidylcholine-induced oxidized LDL uptake in smooth muscle cells. FEBS Lett..

[46] Ross R. (1986). The pathogenesis of atherosclerosis—An update. N. Engl. J. Med..

[47] Lao K.H., Zeng L., Xu Q. (2015). Endothelial and smooth muscle cell transformation in atherosclerosis. Curr. Opin. Lipidol..

[48] Mietus-Snyder M., Gowri M.S., Pitas R.E. (2000). Class A Scavenger Receptor Up-regulation in Smooth Muscle Cells by Oxidized Low Density Lipoprotein*: Enhancement by calcium flux and concurrent cyclooxygenase-2 up-regulation. J. Biol. Chem..

[49] Jalvy S., Renault M.-A., Leen L.L.S., Belloc I., Bonnet J., Gadeau A.-P., Desgranges C. (2007). Autocrine expression of osteopontin contributes to PDGF-mediated arterial smooth muscle cell migration. Cardiovasc. Res..

[50] Raines E.W. (2004). PDGF and cardiovascular disease. Cytokine Growth Factor Rev..

[51] Hegyi L., Skepper J.N., CARY N.R., Mitchinson M.J. (1996). Foam cell apoptosis and the development of the lipid core of human atherosclerosis. J. Pathol..

[52] Kalampogias A., Siasos G., Oikonomou E., Tsalamandris S., Mourouzis K., Tsigkou V., Vavuranakis M., Zografos T., Deftereos S., Stefanadis C. (2016). Basic mechanisms in atherosclerosis: The role of calcium. Med. Chem..

[53] Centelles M.N., Puy C., Lopez-Sagaseta J., Fukudome K., Montes R., Hermida J. (2010). Blocking endothelial protein C receptor (EPCR) accelerates thrombus development in vivo. Thromb. Haemost..

[54] Merlini P.A., Rossi M.L., Faioni E.M., Franchi F., Bramucci E., Lucreziotti S., Biguzzi E., Mannucci P.M., Ardissino D. (2004). Expression of endothelial protein C receptor and thrombomodulin in human coronary atherosclerotic plaques. Ital. Heart J..

[55] Chen P.-S., Wang K.-C., Chao T.-H., Chung H.-C., Tseng S.-Y., Luo C.-Y., Shi G.-Y., Wu H.-L., Li Y.-H. (2017). Recombinant thrombomodulin exerts anti-autophagic action in endothelial cells and provides anti-atherosclerosis effect in apolipoprotein E deficient mice. Sci. Rep..

[56] Stavik B., Holm S., Espada S., Iversen N., Sporsheim B., Bjerkeli V., Dahl T.B., Sandset P.M., Skjelland M., Espevik T. (2017). Increased expression of TFPI in human carotid stenosis. Thromb. Res..

[57] Uszyński M., Żekanowska E., Uszyński W., Kuczyński J. (2001). Tissue factor (TF) and tissue factor pathway inhibitor (TFPI) in amniotic fluid and blood plasma: Implications for the mechanism of amniotic fluid embolism. Eur. J. Obstet. Gynecol. Reprod. Biol..

[58] Smith E.B. (1960). Intimal and medial lipids in human aortas. Lancet.

[59] Edsfeldt A., Dunér P., Ståhlman M., Mollet I.G., Asciutto G., Grufman H., Nitulescu M., Persson A.F., Fisher R.M., Melander O. (2016). Sphingolipids contribute to human atherosclerotic plaque inflammation. Arterioscler. Thromb. Vasc. Biol..

[60] Hornemann T., Worgall T.S. (2013). Sphingolipids and atherosclerosis. Atherosclerosis.

[61] Manicke N.E., Nefliu M., Wu C., Woods J.W., Reiser V., Hendrickson R.C., Cooks R.G. (2009). Imaging of lipids in atheroma by desorption electrospray ionization mass spectrometry. Anal. Chem..

[62] Portman O.W., Alexander M. (1970). Metabolism of sphingolipids by normal and atherosclerotic aorta of squirrel monkeys. J. Lipid Res..

[63] Zheng W., Kollmeyer J., Symolon H., Momin A., Munter E., Wang E., Kelly S., Allegood J.C., Liu Y., Peng Q. (2006). Ceramides and other bioactive sphingolipid backbones in health and disease: Lipidomic analysis, metabolism and roles in membrane structure, dynamics, signaling and autophagy. Biochim. Biophys. Acta.

[64] Gagliostro V., Casas J., Caretti A., Abad J.L., Tagliavacca L., Ghidoni R., Fabrias G., Signorelli P. (2012). Dihydroceramide delays cell cycle G1/S transition via activation of ER stress and induction of autophagy. Int. J. Biochem. Cell Biol..

[65] Signorelli P., Munoz-Olaya J.M., Gagliostro V., Casas J., Ghidoni R., Fabriàs G. (2009). Dihydroceramide intracellular increase in response to resveratrol treatment mediates autophagy in gastric cancer cells. Cancer Lett..

[66] Venant H., Rahmaniyan M., Jones E.E., Lu P., Lilly M.B., Garrett-Mayer E., Drake R.R., Kraveka J.M., Smith C.D., Voelkel-Johnson C. (2015). The Sphingosine Kinase 2 Inhibitor ABC294640 Reduces the Growth of Prostate Cancer Cells and Results in Accumulation of Dihydroceramides In Vitro and In Vivo. Mol. Cancer.

[67] Breen P., Joseph N., Thompson K., Kraveka J.M., Gudz T.I., Li L., Rahmaniyan M., Bielawski J., Pierce J.S., van Buren E. (2013). Dihydroceramide desaturase knockdown impacts sphingolipids and apoptosis after photodamage in human head and neck squamous carcinoma cells. Anticancer Res..

[68] Lachkar F., Ferré P., Foufelle F., Papaioannou A. (2021). Dihydroceramides: Their emerging physiological roles and functions in cancer and metabolic diseases. Am. J. Physiol.-Endocrinol. Metab..

[69] Jiang Q., Rao X., Kim C.Y., Freiser H., Zhang Q., Jiang Z., Li G. (2012). Gamma-tocotrienol induces apoptosis and autophagy in prostate cancer cells by increasing intracellular dihydrosphingosine and dihydroceramide. Int. J. Cancer.

[70] Hassanpour M., Rahbarghazi R., Nouri M., Aghamohammadzadeh N., Safaei N., Ahmadi M. (2019). Role of autophagy in atherosclerosis: Foe or friend?. J. Inflamm..

[71] Stiban J., Fistere D., Colombini M. (2006). Dihydroceramide hinders ceramide channel formation: Implications on apoptosis. Apoptosis.

[72] Magaye R.R., Savira F., Hua Y., Kelly D.J., Reid C., Flynn B., Liew D., Wang B.H. (2019). The role of dihydrosphingolipids in disease. Cell. Mol. Life Sci..

[73] Siddique M.M., Li Y., Chaurasia B., Kaddai V.A., Summers S.A. (2015). Dihydroceramides: From bit players to lead actors. J. Biol. Chem..

[74] Huwiler A., Kolter T., Pfeilschifter J., Sandhoff K. (2000). Physiology and pathophysiology of sphingolipid metabolism and signaling. Biochim. Biophys. Acta.

[75] Augé N., Andrieu N., Nègre-Salvayre A., Thiers J.-C., Levade T., Salvayre R. (1996). The sphingomyelin-ceramide signaling pathway is involved in oxidized low density lipoprotein-induced cell proliferation. J. Biol. Chem..

[76] Poss A.M., Maschek J.A., Cox J.E., Hauner B.J., Hopkins P.N., Hunt S.C., Holland W.L., Summers S.A., Playdon M.C. (2020). Machine learning reveals serum sphingolipids as cholesterol-independent biomarkers of coronary artery disease. J. Clin. Investig..

[77] Laaksonen R., Ekroos K., Sysi-Aho M., Hilvo M., Vihervaara T., Kauhanen D., Suoniemi M., Hurme R., März W., Scharnagl H. (2016). Plasma ceramides predict cardiovascular death in patients with stable coronary artery disease and acute coronary syndromes beyond LDL-cholesterol. Eur. Heart J..

[78] Jiang Z., Hong X., Long H., Hu J. (2000). Ceramides induce apoptosis in HeLa cells and enhance cytochrome c-induced apoptosis in Xenopus egg extracts. Cell Mol. Life Sci..

[79] Schissel S.L., Tweedie-Hardman J., Rapp J.H., Graham G., Williams K.J., Tabas I. (1996). Rabbit aorta and human atherosclerotic lesions hydrolyze the sphingomyelin of retained low-density lipoprotein. Proposed role for arterial-wall sphingomyelinase in subendothelial retention and aggregation of atherogenic lipoproteins. J. Clin. Investig..

[80] Ichi I., Nakahara K., Miyashita Y., Hidaka A., Kutsukake S., Inoue K., Maruyama T., Miwa Y., Harada-Shiba M., Tsushima M. (2006). Association of ceramides in human plasma with risk factors of atherosclerosis. Lipids.

[81] Tomiuk S., Zumbansen M., Stoffel W. (2000). Characterization and subcellular localization of murine and human magnesium-dependent neutral sphingomyelinase. J. Biol. Chem..

[82] Jung S.Y., Suh J.H., Park H.J., Jung K.M., Kim M.Y., Na D.S., Kim D.K. (2000). Identification of Multiple Forms of Membrane-Associated Neutral Sphingomyelinase in Bovine Brain. J. Neurochem..

[83] Casula M., Colpani O., Xie S., Catapano A.L., Baragetti A. (2021). HDL in Atherosclerotic Cardiovascular Disease: In Search of a Role. Cells.

[84] Elshourbagy N.A., Meyers H.V., Abdel-Meguid S.S. (2014). Cholesterol: The good, the bad, and the ugly-therapeutic targets for the treatment of dyslipidemia. Med. Princ. Pract..

[85] Zhong S., Sharp D.S., Grove J.S., Bruce C., Yano K., Curb J.D., Tall A.R. (1996). Increased coronary heart disease in Japanese-American men with mutation in the cholesteryl ester transfer protein gene despite increased HDL levels. J. Clin. Investig..

[86] Kolmakova A., Kwiterovich P., Virgil D., Alaupovic P., Knight-Gibson C., Martin S.F., Chatterjee S. (2004). Apolipoprotein CI induces apoptosis in human aortic smooth muscle cells via recruiting neutral sphingomyelinase. Arterioscler. Thromb. Vasc. Biol..

[87] Devillard R., Galvani S., Thiers J.-C., Guenet J.-L., Hannun Y., Bielawski J., Nègre-Salvayre A., Salvayre R., Augé N. (2010). Stress-induced sphingolipid signaling: Role of type-2 neutral sphingomyelinase in murine cell apoptosis and proliferation. PLoS ONE.

[88] Zettler M.E., Prociuk M.A., Austria J.A., Massaeli H., Zhong G., Pierce G.N. (2003). OxLDL stimulates cell proliferation through a general induction of cell cycle proteins. Am. J. Physiol.-Heart Circ. Physiol..

[89] Marathe S., Choi Y., Leventhal A.R., Tabas I. (2000). Sphingomyelinase converts lipoproteins from apolipoprotein E knockout mice into potent inducers of macrophage foam cell formation. Arterioscler. Thromb. Vasc. Biol..

[90] Devlin C.M., Leventhal A.R., Kuriakose G., Schuchman E.H., Williams K.J., Tabas I. (2008). Acid sphingomyelinase promotes lipoprotein retention within early atheromata and accelerates lesion progression. Arterioscler. Thromb. Vasc. Biol..

[91] Pavoine C., Pecker F. (2009). Sphingomyelinases: Their regulation and roles in cardiovascular pathophysiology. Cardiovasc. Res..

[92] Goñi F.M., Alonso A. (2002). Sphingomyelinases: Enzymology and membrane activity. FEBS Lett..

[93] Marchesini N., Hannun Y.A. (2004). Acid and neutral sphingomyelinases: Roles and mechanisms of regulation. Biochem. Cell Biol..

[94] Grassme H., Jekle A., Riehle A., Schwarz H., Berger J., Sandhoff K., Kolesnick R., Gulbins E. (2001). CD95 signaling via ceramide-rich membrane rafts. J. Biol. Chem..

[95] Jin S., Yi F., Zhang F., Poklis J.L., Li P.-L. (2008). Lysosomal targeting and trafficking of acid sphingomyelinase to lipid raft platforms in coronary endothelial cells. Arterioscler. Thromb. Vasc. Biol..

[96] Zhang A.Y., Yi F., Jin S., Xia M., Chen Q.Z., Gulbins E., Li P.L. (2007). Acid sphingomyelinase and its redox amplification in formation of lipid raft redox signaling platforms in endothelial cells. Antioxid. Redox Signal..

[97] Jia S.-J., Jin S., Zhang F., Yi F., Dewey W.L., Li P.-L. (2008). Formation and function of ceramide-enriched membrane platforms with CD38 during M1-receptor stimulation in bovine coronary arterial myocytes. Am. J. Physiol.-Heart Circ. Physiol..

[98] Augé N., Maupas-Schwalm F., Elbaz M., Thiers J.-C., Waysbort A., Itohara S., Krell H.-W., Salvayre R., Nègre-Salvayre A. (2004). Role for Matrix Metalloproteinase-2 in Oxidized Low-Density Lipoprotein–Induced Activation of the Sphingomyelin/Ceramide Pathway and Smooth Muscle Cell Proliferation. Circulation.

[99] Ikeda U., Shimada K. (2003). Matrix metalloproteinases and coronary artery diseases. Clin. Cardiol. Int. Index. Peer-Rev. J. Adv. Treat. Cardiovasc. Dis..

[100] Galis Z.S., Khatri J.J. (2002). Matrix metalloproteinases in vascular remodeling and atherogenesis: The good, the bad, and the ugly. Circ. Res..

[101] Xu J., Yeh C.-H., Chen S., He L., Sensi S.L., Canzoniero L.M., Choi D.W., Hsu C.Y. (1998). Involvement of de NovoCeramide Biosynthesis in Tumor Necrosis Factor-α/Cycloheximide-induced Cerebral Endothelial Cell Death. J. Biol. Chem..

[102] Modur V., Zimmerman G.A., Prescott S.M., McIntyre T.M. (1996). Endothelial cell inflammatory responses to tumor necrosis factor α: Ceramide-dependent and-independent mitogen-activated protein kinase cascades. J. Biol. Chem..

[103] Bhagat K., Vallance P. (1997). Inflammatory cytokines impair endothelium-dependent dilatation in human veins in vivo. Circulation.

[104] Nakamura M., Yoshida H., Arakawa N., Saitoh S., Satoh M., Hiramori K. (2000). Effects of tumor necrosis factor-α on basal and stimulated endothelium-dependent vasomotion in human resistance vessel. J. Cardiovasc. Pharmacol..

[105] Wang P., Ba Z.F., Chaudry I.H. (1994). Administration of tumor necrosis factor-alpha in vivo depresses endothelium-dependent relaxation. Am. J. Physiol.-Heart Circ. Physiol..

[106] Zhang H., Park Y., Wu J., Chen X.P., Lee S., Yang J., Dellsperger K.C., Zhang C. (2009). Role of TNF-α in vascular dysfunction. Clin. Sci..

[107] Sawada M., Kiyono T., Nakashima S., Shinoda J., Naganawa T., Hara S., Iwama T., Sakai N. (2004). Molecular mechanisms of TNF-α-induced ceramide formation in human glioma cells: p53-mediated oxidant stress-dependent and-independent pathways. Cell Death Differ..

[108] Zhang D.X., Zou A.-P., Li P.-L. (2003). Ceramide-induced activation of NADPH oxidase and endothelial dysfunction in small coronary arteries. Am. J. Physiol.-Heart Circ. Physiol..

[109] Zhang D.X., Fryer R.M., Hsu A.K., Zou A.-P., Gross G.J., Campbell W.B., Li P.-L. (2001). Production and metabolism of ceramide in normal and ischemic-reperfused myocardium of rats. Basic Res. Cardiol..

[110] Hirokawa M., Kitabayashi A., Kuroki J., Miura A.B. (2000). Induction of tissue factor production but not the upregulation of adhesion molecule expression by ceramide in human vascular endothelial cells. Tohoku J. Exp. Med..

[111] Ito H., Koide N., Hassan F., Islam S., Tumurkhuu G., Mori I., Yoshida T., Kakumu S., Moriwaki H., Yokochi T. (2006). Lethal endotoxic shock using α-galactosylceramide sensitization as a new experimental model of septic shock. Lab. Investig..

[112] Hürlimann D., Forster A., Noll G., Enseleit F., Chenevard R., Distler O., Béchir M., Spieker L.E., Neidhart M., Michel B.A. (2002). Anti–tumor necrosis factor-α treatment improves endothelial function in patients with rheumatoid arthritis. Circulation.

[113] Yang Z.-Z., Zou A.-P. (2003). Homocysteine enhances TIMP-1 expression and cell proliferation associated with NADH oxidase in rat mesangial cells. Kidney Int..

[114] Yi F., Zhang A.Y., Janscha J.L., Li P.-L., Zou A.-P. (2004). Homocysteine activates NADH/NADPH oxidase through ceramide-stimulated Rac GTPase activity in rat mesangial cells. Kidney Int..

[115] Bulotta S., Barsacchi R., Rotiroti D., Borgese N., Clementi E. (2001). Activation of the endothelial nitric-oxide synthase by tumor necrosis factor-α: A novel feedback mechanism regulating cell death. J. Biol. Chem..

[116] Igarashi J., Thatte H.S., Prabhakar P., Golan D.E., Michel T. (1999). Calcium-independent activation of endothelial nitric oxide synthase by ceramide. Proc. Natl. Acad. Sci. USA.

[117] Li H., Junk P., Huwiler A., Burkhardt C., Wallerath T., Pfeilschifter J., Förstermann U. (2002). Dual effect of ceramide on human endothelial cells: Induction of oxidative stress and transcriptional upregulation of endothelial nitric oxide synthase. Circulation.

[118] Bhunia A.K., Han H., Snowden A., Chatterjee S. (1997). Redox-regulated signaling by lactosylceramide in the proliferation of human aortic smooth muscle cells. J. Biol. Chem..

[119] Harada-Shiba M., Kinoshita M., Kamido H., Shimokado K. (1998). Oxidized low density lipoprotein induces apoptosis in cultured human umbilical vein endothelial cells by common and unique mechanisms. J. Biol. Chem..

[120] Ruuth M., Nguyen S.D., Vihervaara T., Hilvo M., Laajala T.D., Kondadi P.K., Gisterå A., Lähteenmäki H., Kittilä T., Huusko J. (2018). Susceptibility of low-density lipoprotein particles to aggregate depends on particle lipidome, is modifiable, and associates with future cardiovascular deaths. Eur. Heart J..

[121] Morita S.-Y., Kawabe M., Sakurai A., Okuhira K., Vertut-Doï A., Nakano M., Handa T. (2004). Ceramide in lipid particles enhances heparan sulfate proteoglycan and low density lipoprotein receptor-related protein-mediated uptake by macrophages. J. Biol. Chem..

[122] Morita S.-Y., Nakano M., Sakurai A., Deharu Y., Vertut-Doï A., Handa T. (2005). Formation of ceramide-enriched domains in lipid particles enhances the binding of apolipoprotein E. FEBS Lett..

[123] Öörni K., Hakala J.K., Annila A., Ala-Korpela M., Kovanen P.T. (1998). Sphingomyelinase induces aggregation and fusion, but phospholipase A2 only aggregation, of low density lipoprotein (LDL) particles: Two distinct mechanisms leading to increased binding strength of LDL to human aortic proteoglycans. J. Biol. Chem..

[124] Zelnik I.D., Ventura A.E., Kim J.L., Silva L.C., Futerman A.H. (2020). The role of ceramide in regulating endoplasmic reticulum function. Biochim. Et Biophys. Acta (BBA)-Mol. Cell Biol. Lipids.

[125] Sneck M., Nguyen S.D., Pihlajamaa T., Yohannes G., Riekkola M.-L., Milne R., Kovanen P.T., Öörni K. (2012). Conformational changes of apoB-100 in SMase-modified LDL mediate formation of large aggregates at acidic pH [S]. J. Lipid Res..

[126] Benitez-Amaro A., Pallara C., Nasarre L., Rivas-Urbina A., Benitez S., Vea A., Bornachea O., de Gonzalo-Calvo D., Serra-Mir G., Villegas S. (2019). Molecular basis for the protective effects of low-density lipoprotein receptor-related protein 1 (LRP1)-derived peptides against LDL aggregation. Biochim. Biophys. Acta (BBA)-Biomembr..

[127] Hojjati M.R., Li Z., Zhou H., Tang S., Huan C., Ooi E., Lu S., Jiang X.-C. (2005). Effect of myriocin on plasma sphingolipid metabolism and atherosclerosis in apoE-deficient mice. J. Biol. Chem..

[128] Hartmann D., Lucks J., Fuchs S., Schiffmann S., Schreiber Y., Ferreirós N., Merkens J., Marschalek R., Geisslinger G., Grösch S. (2012). Long chain ceramides and very long chain ceramides have opposite effects on human breast and colon cancer cell growth. Int. J. Biochem. Cell Biol..

[129] Lallemand T., Rouahi M., Swiader A., Grazide M.-H., Geoffre N., Alayrac P., Recazens E., Coste A., Salvayre R., Nègre-Salvayre A. (2018). nSMase2 (type 2-neutral sphingomyelinase) deficiency or inhibition by GW4869 reduces inflammation and atherosclerosis in Apoe−/−mice. Arterioscler. Thromb. Vasc. Biol..

[130] Law B.A., Liao X., Moore K.S., Southard A., Roddy P., Ji R., Szulc Z., Bielawska A., Schulze P.C., Cowart L.A. (2018). Lipotoxic very-long-chain ceramides cause mitochondrial dysfunction, oxidative stress, and cell death in cardiomyocytes. FASEB J..

[131] Stunff H.L., Milstien S., Spiegel S. (2004). Generation and metabolism of bioactive sphingosine-1-phosphate. J. Cell. Biochem..

[132] Yatomi Y., Ruan F., Hakomori S.-I., Igarashi Y. (1995). Sphingosine-1-phosphate: A platelet-activating sphingolipid released from agonist-stimulated human platelets. Blood.

[133] Pappu R., Schwab S.R., Cornelissen I., Pereira J.P., Regard J.B., Xu Y., Camerer E., Zheng Y.-W., Huang Y., Cyster J.G. (2007). Promotion of lymphocyte egress into blood and lymph by distinct sources of sphingosine-1-phosphate. Science.

[134] Venkataraman K., Lee Y.-M., Michaud J., Thangada S., Ai Y., Bonkovsky H.L., Parikh N.S., Habrukowich C., Hla T. (2008). Vascular endothelium as a contributor of plasma sphingosine 1-phosphate. Circ. Res..

[135] Fukuhara S., Simmons S., Kawamura S., Inoue A., Orba Y., Tokudome T., Sunden Y., Arai Y., Moriwaki K., Ishida J. (2012). The sphingosine-1-phosphate transporter Spns2 expressed on endothelial cells regulates lymphocyte trafficking in mice. J. Clin. Investig..

[136] Simmons S., Sasaki N., Umemoto E., Uchida Y., Fukuhara S., Kitazawa Y., Okudaira M., Inoue A., Tohya K., Aoi K. (2019). High-endothelial cell-derived S1P regulates dendritic cell localization and vascular integrity in the lymph node. Elife.

[137] Vu T.M., Ishizu A.N., Foo J.C., Toh X.R., Zhang F., Whee D.M., Torta F., Cazenave-Gassiot A., Matsumura T., Kim S. (2017). Mfsd2b is essential for the sphingosine-1-phosphate export in erythrocytes and platelets. Nature.

[138] Kobayashi N., Kawasaki-Nishi S., Otsuka M., Hisano Y., Yamaguchi A., Nishi T. (2018). MFSD2B is a sphingosine 1-phosphate transporter in erythroid cells. Sci. Rep..

[139] Yanagida K., Hla T. (2017). Vascular and Immunobiology of the Circulatory Sphingosine 1-Phosphate Gradient. Annu. Rev. Physiol..

[140] Deutschman D.H., Carstens J.S., Klepper R.L., Smith W.S., Page M.T., Young T.R., Gleason L.A., Nakajima N., Sabbadini R.A. (2003). Predicting obstructive coronary artery disease with serum sphingosine-1-phosphate. Am. Heart J..

[141] Brinkmann V., Billich A., Baumruker T., Heining P., Schmouder R., Francis G., Aradhye S., Burtin P. (2010). Fingolimod (FTY720): Discovery and development of an oral drug to treat multiple sclerosis. Nat. Rev. Drug Discov..

[142] Keul P., Sattler K., Levkau B. (2007). HDL and its sphingosine-1-phosphate content in cardioprotection. Heart Fail. Rev..

[143] Nofer J.R., Bot M., Brodde M., Taylor P.J., Salm P., Brinkmann V., van Berkel T., Assmann G., Biessen E.A. (2007). FTY720, a synthetic sphingosine 1 phosphate analogue, inhibits development of atherosclerosis in low-density lipoprotein receptor-deficient mice. Circulation.

[144] Xu N., Dahlbäck B. (1999). A novel human apolipoprotein (apoM). J. Biol. Chem..

[145] Christoffersen C., Obinata H., Kumaraswamy S.B., Galvani S., Ahnström J., Sevvana M., Egerer-Sieber C., Muller Y.A., Hla T., Nielsen L.B. (2011). Endothelium-protective sphingosine-1-phosphate provided by HDL-associated apolipoprotein M. Proc. Natl. Acad. Sci. USA.

[146] Aoki S., Yatomi Y., Ohta M., Osada M., Kazama F., Satoh K., Nakahara K., Ozaki Y. (2005). Sphingosine 1-phosphate-related metabolism in the blood vessel. J. Biochem..

[147] Ohkawa R., Nakamura K., Okubo S., Hosogaya S., Ozaki Y., Tozuka M., Osima N., Yokota H., Ikeda H., Yatomi Y. (2008). Plasma sphingosine-1-phosphate measurement in healthy subjects: Close correlation with red blood cell parameters. Ann. Clin. Biochem..

[148] Zhang B., Tomura H., Kuwabara A., Kimura T., Miura S., Noda K., Okajima F., Saku K. (2005). Correlation of high density lipoprotein (HDL)-associated sphingosine 1-phosphate with serum levels of HDL-cholesterol and apolipoproteins. Atherosclerosis.

[149] Levkau B. (2015). HDL-S1P: Cardiovascular functions, disease-associated alterations, and therapeutic applications. Front. Pharmacol..

[150] Kurano M., Tsukamoto K., Hara M., Ohkawa R., Ikeda H., Yatomi Y. (2015). LDL receptor and ApoE are involved in the clearance of ApoM-associated sphingosine 1-phosphate. J. Biol. Chem..

[151] Kappelle P.J., Ahnström J., Dikkeschei B.D., de Vries R., Sluiter W.J., Wolffenbuttel B.H., van Tol A., Nielsen L.B., Dahlbäck B., Dullaart R.P. (2010). Plasma apolipoprotein M responses to statin and fibrate administration in type 2 diabetes mellitus. Atherosclerosis.

[152] Galvani S., Sanson M., Blaho V.A., Swendeman S.L., Obinata H., Conger H., Dahlbäck B., Kono M., Proia R.L., Smith J.D. (2015). HDL-bound sphingosine 1-phosphate acts as a biased agonist for the endothelial cell receptor S1P1 to limit vascular inflammation. Sci. Signal..

[153] Kurano M., Yatomi Y. (2017). Sphingosine 1-phosphate and atherosclerosis. J. Atheroscler. Thromb..

[154] Argraves K.M., Gazzolo P.J., Groh E.M., Wilkerson B.A., Matsuura B.S., Twal W.O., Hammad S.M., Argraves W.S. (2008). High density lipoprotein-associated sphingosine 1-phosphate promotes endothelial barrier function. J. Biol. Chem..

[155] Filep J.G., Földes-Filep É., Sirois P. (1993). Nitric oxide modulates vascular permeability in the rat coronary circulation. Br. J. Pharmacol..

[156] Nofer J.-R., Van Der Giet M., Tölle M., Wolinska I., von Wnuck Lipinski K., Baba H.A., Tietge U.J., Gödecke A., Ishii I., Kleuser B. (2004). HDL induces NO-dependent vasorelaxation via the lysophospholipid receptor S1P 3. J. Clin. Investig..

[157] Kimura T., Tomura H., Mogi C., Kuwabara A., Damirin A., Ishizuka T., Sekiguchi A., Ishiwara M., Im D.-S., Sato K. (2006). Role of scavenger receptor class B type I and sphingosine 1-phosphate receptors in high density lipoprotein-induced inhibition of adhesion molecule expression in endothelial cells. J. Biol. Chem..

[158] Ruiz M., Frej C., Holmér A., Guo L.J., Tran S., Dahlbäck B. (2017). High-density lipoprotein–associated apolipoprotein M limits endothelial inflammation by delivering sphingosine-1-phosphate to the sphingosine-1-phosphate receptor 1. Arterioscler. Thromb. Vasc. Biol..

[159] Theilmeier G., Schmidt C., Herrmann J.R., Keul P., Schäfers M., Herrgott I., Mersmann J., Larmann J., Hermann S., Stypmann J.R. (2006). High-density lipoproteins and their constituent, sphingosine-1-phosphate, directly protect the heart against ischemia/reperfusion injury in vivo via the S1P3 lysophospholipid receptor. Circulation.

[160] Keul P., Lucke S., von Wnuck Lipinski K., Bode C., Gräler M., Heusch G., Levkau B. (2011). Sphingosine-1-phosphate receptor 3 promotes recruitment of monocyte/macrophages in inflammation and atherosclerosis. Circ. Res..

[161] Tamama K., Tomura H., Sato K., Malchinkhuu E., Damirin A., Kimura T., Kuwabara A., Murakami M., Okajima F. (2005). High-density lipoprotein inhibits migration of vascular smooth muscle cells through its sphingosine 1-phosphate component. Atherosclerosis.

[162] Duenas A.I., Aceves M., Fernández-Pisonero I., Gómez C., Orduna A., Crespo M.S., García-Rodríguez C. (2008). Selective attenuation of Toll-like receptor 2 signalling may explain the atheroprotective effect of sphingosine 1-phosphate. Cardiovasc. Res..

[163] Skoura A., Michaud J., Im D.-S., Thangada S., Xiong Y., Smith J.D., Hla T. (2011). Sphingosine-1-phosphate receptor-2 function in myeloid cells regulates vascular inflammation and atherosclerosis. Arterioscler. Thromb. Vasc. Biol..

[164] Sanchez T., Skoura A., Wu M.T., Casserly B., Harrington E.O., Hla T. (2007). Induction of vascular permeability by the sphingosine-1-phosphate receptor–2 (S1P2R) and its downstream effectors ROCK and PTEN. Arterioscler. Thromb. Vasc. Biol..

[165] MacLennan A.J., Carney P.R., Zhu W.J., Chaves A.H., Garcia J., Grimes J.R., Anderson K.J., Roper S.N., Lee N. (2001). An essential role for the H218/AGR16/Edg-5/LPB2 sphingosine 1-phosphate receptor in neuronal excitability. Eur. J. Neurosci..

[166] Cattoretti G., Mandelbaum J., Lee N., Chaves A.H., Mahler A.M., Chadburn A., Dalla-Favera R., Pasqualucci L., MacLennan A.J. (2009). Targeted disruption of the S1P2 sphingosine 1-phosphate receptor gene leads to diffuse large B-cell lymphoma formation. Cancer Res..

[167] Green J.A., Suzuki K., Cho B., Willison L.D., Palmer D., Allen C.D., Schmidt T.H., Xu Y., Proia R.L., Coughlin S.R. (2011). The sphingosine 1-phosphate receptor S1P 2 maintains the homeostasis of germinal center B cells and promotes niche confinement. Nat. Immunol..

[168] Rivera J., Proia R.L., Olivera A. (2008). The alliance of sphingosine-1-phosphate and its receptors in immunity. Nat. Rev. Immunol..

[169] Terai K., Soga T., Takahashi M., Kamohara M., Ohno K., Yatsugi S., Okada M., Yamaguchi T. (2003). Edg-8 receptors are preferentially expressed in oligodendrocyte lineage cells of the rat CNS. Neuroscience.

[170] Wang W., Graeler M.H., Goetzl E.J. (2005). Type 4 sphingosine 1-phosphate G protein-coupled receptor (S1P4) transduces S1P effects on T cell proliferation and cytokine secretion without signaling migration. FASEB J..

[171] Slotte P.J. (2013). Molecular properties of various structurally defined sphingomyelins–correlation of structure with function. Prog. Lipid Res..

[172] Patton S. (1970). Correlative relationship of cholesterol and sphingomyelin in cell membranes. J. Theor. Biol..

[173] Slotte J.P. (2013). Biological functions of sphingomyelins. Prog. Lipid Res..

[174] Jeong T., Schissel S.L., Tabas I., Pownall H.J., Tall A.R., Jiang X.-C. (1998). Increased sphingomyelin content of plasma lipoproteins in apolipoprotein E knockout mice reflects combined production and catabolic defects and enhances reactivity with mammalian sphingomyelinase. J. Clin. Investig..

[175] Bojic L.A., McLaren D.G., Shah V., Previs S.F., Johns D.G., Castro-Perez J.M. (2014). Lipidome of atherosclerotic plaques from hypercholesterolemic rabbits. Int. J. Mol. Sci..

[176] Camont L., Lhomme M., Rached F., Le Goff W., Nègre-Salvayre A., Salvayre R., Calzada C., Lagarde M., Chapman M.J., Kontush A. (2013). Small, dense high-density lipoprotein-3 particles are enriched in negatively charged phospholipids: Relevance to cellular cholesterol efflux, antioxidative, antithrombotic, anti-inflammatory, and antiapoptotic functionalities. Arterioscler. Thromb. Vasc. Biol..

[177] Mäkinen V.-P., Tynkkynen T., Soininen P., Forsblom C., Peltola T., Kangas A.J., Groop P.-H., Ala-Korpela M. (2012). Sphingomyelin is associated with kidney disease in type 1 diabetes (The FinnDiane Study). Metabolomics.

[178] Adachi R., Ogawa K., Matsumoto S.-i., Satou T., Tanaka Y., Sakamoto J., Nakahata T., Okamoto R., Kamaura M., Kawamoto T. (2017). Discovery and characterization of selective human sphingomyelin synthase 2 inhibitors. Eur. J. Med. Chem..

[179] Yu Z., Peng Q., Huang Y. (2019). Potential therapeutic targets for atherosclerosis in sphingolipid metabolism. Clin. Sci..

[180] Li Z., Fan Y., Liu J., Li Y., Huan C., Bui H.H., Kuo M.S., Park T.S., Cao G., Jiang X.C. (2012). Impact of sphingomyelin synthase 1 deficiency on sphingolipid metabolism and atherosclerosis in mice. Arter. Thromb. Vasc. Biol..

[181] Liu J., Huan C., Chakraborty M., Zhang H., Lu D., Kuo M.-S., Cao G., Jiang X.-C. (2009). Macrophage sphingomyelin synthase 2 deficiency decreases atherosclerosis in mice. Circ. Res..

[182] Wang X., Dong J., Zhao Y., Li Y., Wu M. (2011). Adenovirus-mediated sphingomyelin synthase 2 increases atherosclerotic lesions in ApoE KO mice. Lipids Health Dis..

[183] Hailemariam T.K., Huan C., Liu J., Li Z., Roman C., Kalbfeisch M., Bui H.H., Peake D.A., Kuo M.-S., Cao G. (2008). Sphingomyelin synthase 2 deficiency attenuates NFκB activation. Arterioscler. Thromb. Vasc. Biol..

[184] Dong J., Liu J., Lou B., Li Z., Ye X., Wu M., Jiang X.-C. (2006). Adenovirus-mediated overexpression of sphingomyelin synthases 1 and 2 increases the atherogenic potential in mice. J. Lipid Res..

[185] Lu M.H., Takemoto M., Watanabe K., Luo H., Nishimura M., Yano M., Tomimoto H., Okazaki T., Oike Y., Song W.J. (2012). Deficiency of sphingomyelin synthase-1 but not sphingomyelin synthase-2 causes hearing impairments in mice. J. Physiol..

[186] Yano M., Watanabe K., Yamamoto T., Ikeda K., Senokuchi T., Lu M., Kadomatsu T., Tsukano H., Ikawa M., Okabe M. (2011). Mitochondrial dysfunction and increased reactive oxygen species impair insulin secretion in sphingomyelin synthase 1-null mice. J. Biol. Chem..

[187] Dong L., Watanabe K., Itoh M., Huan C.-R., Tong X.-P., Nakamura T., Miki M., Iwao H., Nakajima A., Sakai T. (2012). CD4+ T-cell dysfunctions through the impaired lipid rafts ameliorate concanavalin A-induced hepatitis in sphingomyelin synthase 1-knockout mice. Int. Immunol..

[188] Chatterjee S., Ghosh N. (1996). Oxidized low density lipoprotein stimulates aortic smooth muscle cell proliferation. Glycobiology.

[189] Chatterjee S., Bedja D., Mishra S., Amuzie C., Avolio A., Kass D.A., Berkowitz D., Renehan M. (2014). Inhibition of glycosphingolipid synthesis ameliorates atherosclerosis and arterial stiffness in apolipoprotein E−/−mice and rabbits fed a high-fat and-cholesterol diet. Circulation.

[190] Arai T., Bhunia A.K., Chatterjee S., Bulkley G.B. (1998). Lactosylceramide stimulates human neutrophils to upregulate Mac-1, adhere to endothelium, and generate reactive oxygen metabolites in vitro. Circ. Res..

[191] Bhunia A.K., Han H., Snowden A., Chatterjee S. (1996). Lactosylceramide Stimulates Ras-GTP Loading, Kinases (MEK, Raf), p44 Mitogen-activated Protein Kinase, and c-fos Expression in Human Aortic Smooth Muscle Cells (*). J. Biol. Chem..

[192] Chatterjee S.B., Dey S., Shi W.Y., Thomas K., Hutchins G.M. (1997). Accumulation of glycosphingolipids in human atherosclerotic plaque and unaffected aorta tissues. Glycobiology.

[193] Chatterjee S. (1991). Lactosylceramide stimulates aortic smooth muscle cell proliferation. Biochem. Biophys. Res. Commun..

[194] Chatterjee S. (1998). Sphingolipids in atherosclerosis and vascular biology. Arterioscler. Thromb. Vasc. Biol..

[195] Sundaresan M., Yu Z.-X., Ferrans V.J., Irani K., Finkel T. (1995). Requirement for generation of H_2_O_2_ for platelet-derived growth factor signal transduction. Science.

[196] Bietrix F., Lombardo E., van Roomen C.P., Ottenhoff R., Vos M., Rensen P.C., Verhoeven A.J., Aerts J.M., Groen A.K. (2010). Inhibition of glycosphingolipid synthesis induces a profound reduction of plasma cholesterol and inhibits atherosclerosis development in APOE* 3 Leiden and low-density lipoprotein receptor−/−mice. Arterioscler. Thromb. Vasc. Biol..

[197] Bhunia A.K., Arai T., Bulkley G., Chatterjee S. (1998). Lactosylceramide mediates tumor necrosis factor-α-induced intercellular adhesion molecule-1 (ICAM-1) expression and the adhesion of neutrophil in human umbilical vein endothelial cells. J. Biol. Chem..

